# Supplementary material for: Active site specificity profiling datasets of matrix metalloproteinases (MMPs) 1, 2, 3, 7, 8, 9, 12, 13 and 14
Source: Data Brief. 2016 Feb 22;7:299–310. doi: 10.1016/j.dib.2016.02.036 (PMC4777984; doi:10.1016/j.dib.2016.02.036)
Supplement: Supplementary file 10 — Supplementary material [file mmc10.zip › WebPICS_hMMP9_T_1%/P3prime.html]

 

PICS results


|  |  |
| --- | --- |
| **P3prime\_A**  31 in 138 sites   22.5 %    effects > 10 perc. pnts.   (vice-versa in brackets)  P1\_A: 12.0 (13.2) |  |
| **P3prime\_G**  35 in 138 sites   25.4 %    effects > 10 perc. pnts.   (vice-versa in brackets)  P2prime\_L: 14.8 (34.6) |  |
| **P3prime\_H**  6 in 138 sites   4.3 %    effects > 10 perc. pnts.   (vice-versa in brackets)  P2\_F: 12.4 (12.4)   P2\_H: 12.4 (12.4)   P1\_H: 13.8 (20.7)   P1\_P: 24.6 (12.4)   P1\_W: 14.5 (29.0)   P1prime\_Q: 26.8 (17.9)   P1prime\_W: 14.5 (29.0)   P2prime\_T: 27.5 (20.7) |  |
| **P3prime\_N**  7 in 138 sites   5.1 %    effects > 10 perc. pnts.   (vice-versa in brackets)  P2\_F: 24.3 (28.2)   P2\_M: 22.1 (17.1)   P1prime\_V: 19.9 (11.6)   P2prime\_I: 32.0 (14.9) |  |
| **P3prime\_S**  23 in 138 sites   16.7 %    effects > 10 perc. pnts.   (vice-versa in brackets)  P1\_E: 10.2 (23.3) |  |
